# Reactive Magnetron Plasma Modification of Electrospun PLLA Scaffolds with Incorporated Chloramphenicol for Controlled Drug Release

**DOI:** 10.3390/polym14030373

**Published:** 2022-01-18

**Authors:** Apollinariya A. Volokhova, Dmitry A. Fedorishin, Arina O. Khvastunova, Tatiana I. Spiridonova, Anna I. Kozelskaya, Julia Kzhyshkowska, Sergei I. Tverdokhlebov, Irina Kurzina

**Affiliations:** 1 Department of Translational Cellular and Molecular Biomedicine, Chemical Faculty, National Research Tomsk State University, 36 Lenin Avenue, 634050 Tomsk, Russia; rapollinariya@gmail.com (A.A.V.); strix187@yandex.ru (D.A.F.); arinafayt@gmail.com (A.O.K.); Julia.kzhyshkowska@medma.uni-heidelberg.de (J.K.); 2 The Weinberg Research Center, National Research Tomsk Polytechnic University, 30 Lenin Avenue, 634050 Tomsk, Russia; spiridonovatis2@gmail.com (T.I.S.); kozelskayaai@tpu.ru (A.I.K.); 3 Medical Faculty Mannheim, Institute of Transfusion Medicine and Immunology, University of Heidelberg, Ludolf-Krehl Street 13-17, 68167 Mannheim, Germany

**Keywords:** controlled drug release, magnetron sputtering, biodegradable polymers, polylactic acid, electrospinning, chloramphenicol

## Abstract

Surface modification with the plasma of the direct current reactive magnetron sputtering has demonstrated its efficacy as a tool for enhancing the biocompatibility of polymeric electrospun scaffolds. Improvement of the surface wettability of materials with water, as well as the formation of active chemical bonds in the near-surface layers, are the main reasons for the described effect. These surface effects are also known to increase the release rate of drugs incorporated in fibers. Herein, we investigated the effect of plasma modification on the chloramphenicol release from electrospun poly (lactic acid) fibrous scaffolds. Scaffolds with high—50 wt./wt.%—drug content were obtained. It was shown that plasma modification leads to an increase in the drug release rate and drug diffusion coefficient, while not deteriorating surface morphology and mechanical properties of scaffolds. The materials’ antibacterial activity was observed to increase in the first day of the experiment, while remaining on the same level as the unmodified group during the next six days. The proposed technique for modifying the surface of scaffolds will be useful for obtaining drug delivery systems with controlled accelerated release, which can expand the possibilities of local applications of antibiotics and other drugs.

## 1. Introduction

Surgical site infections (SSIs) are one of the serious threats associated with the installation of implants during surgery. The causative agents of SSI are most often the Gram-positive bacteria, *Staphylococcus aureus, Staphylococcus epidermidis,* and *Streptococcus* spp., etc. [1] These kinds of bacteria can form harmful and toxic biofilms on the surface of implants [2]. Biofilms are a community of microorganisms embedded in the matrix of an extracellular polymeric substance. Biofilms form on the surface of the implant or at the interface between living tissue and dead bone, preventing the bone healing process. Subpopulations of bacteria in the biofilm can also differentiate into a phenotypically stable state and form biofilm-specific antimicrobial resistant genes, which significantly reduces the effectiveness of antibiotic therapy [2]. The combination of these factors can further lead to the rejection of the implant by the body, sepsis, and the need for reoperation [2].

Systemic antibiotic therapy is widely used for the prevention and treatment of SSI. Despite the proven effectiveness, this approach has a number of negative consequences: toxic effects on non-targeted organs and tissues, the complexity of drug delivery to the surgical site, the development of antibiotic resistance, etc. [3].

Local drug delivery appears to be a more promising solution for preventing complications associated with SSIs. This approach provides the ability to achieve high local concentrations in excess of the minimum inhibitory concentration (MIC) required to suppress bacteria with both sustained delivery over determined period of time and targeted delivery [4]. Systemic concentrations, high enough to exert excessive toxic effects on the body, could be avoided with this approach, which increases patient compliance [5]. For controlled release systems, an initial large amount of drug is often released immediately upon placement in the release medium, and it is only after this stage that the release rate profile becomes stable. This phenomenon is typically referred to by the term “burst release”, and is mostly considered as negative [6]. However, in some cases, high initial drug concentration is an important part of the drug administration strategy, antibiotics being one of them. Moreover, long-term achievement of the minimum inhibitory/effective concentration of antibiotic and chemotherapeutical agents can critically affect the effectiveness of therapy [7].

During the past decades, biodegradable electrospun polymeric scaffolds have been found to be of particular interest because they possess high surface area-to-volume ratio, which is crucial not only for the materials applied for tissue engineering purposes [8], but also as drug delivery systems (DDS) [9,10,11]. Several natural [12] and synthetic [13,14] antibiotics were effectively incorporated into electrospun scaffolds for creating sustained release drug delivery devices. 

Chloramphenicol is an antibiotic used for the treatment of bacterial infections. However, for past decades, its use has been limited due to high systemic toxicity. New polymer-based carriers, such as liposomes [15], were proposed to eliminate those negative effects. Several studies where the drug was incorporated into the structure of electrospun scaffolds have been published [16,17,18]. The materials were studied as potential candidates for wound dressings, and their antibacterial properties were demonstrated.

Poly-L-lactic acid (PLLA) is a widely-used bioresorbable, biodegradable polymer for DDS production because of its biocompatibility, the ease of the material for biomedical applications fabrication, and its appropriate mechanical properties [19]. PLLA gradually degrades by hydrolysis and enzymatic processes to non-toxic products, which are completely eliminated from the body. PLLA films with up to 30 wt./wt.% chloramphenicol concentration have successfully been obtained [16]. 

It is known that material surface energy and wettability are some of the key features responsible not only for scaffolds’ biocompatibility, but also for drug release profile. PLLA as poly(ε-caprolactone), poly (vinyl alcohol), and most of the polymers commonly used for medical devices are highly hydrophobic. 

Plasma treatment is one of the most widely-accepted methods to improve the wettability of biomedical polymers [20,21]. Affecting the surface layer of a polymer without changing its bulk properties, production of the continuous and homogeneous coatings, the achievement of the desired chemical modification by means of various treatment parameters, adaptability for all polymers, and dry technology are the main advantages of using a plasma technique [22,23,24]. The electrons, ions, molecules, and other species present in plasma interact with the thin polymer surface, which leads to its chemical functionalization by different chemical groups and/or surface etching [23]. Other effects such as cleaning, activation, and cross-linking can be observed after plasma treatment [25,26]. The effect of plasma modification depends on plasma sources (gas discharge) and plasma treatment parameters (working gas, temperature, pressure in the chamber, power density, treatment duration, etc.).

The simplest and most economical approach, which does not require expensive vacuum equipment, is the treatment of polymers in atmospheric plasma. One of the easily ignited discharges at atmospheric pressure is dielectric barrier discharge. Another two examples of the non-thermal atmospheric pressure plasma sources used for polymer treatment are corona discharge [27] and atmospheric pressure gliding arc [28]. Corona discharge treatment is very similar to atmospheric-pressure plasma. The ignition of plasma occurs in the space between two electrodes surrounded by gas. However, corona discharge treatment is associated with lower overall plasma density, which results in decreasing the rate and degree of the ionization of the molecules. The gliding arc is a promising method for the quick and efficient plasma treatment of the polymer surface due to a high concentration of reactive radicals available for the treatment [29].

However, the treatment in atmospheric plasma has limitations associated with plasma treatment of oxidation-sensitive objects, difficulty in adjusting the parameters of the processes in order to avoid combustion of the material due to the high temperature of the plasma jet (200–300 °C), and difficulty in the formation highly homogenous coatings [30]. Most researchers prefer to use low pressure plasma (glow discharge, the direct current (DC) and radio frequency (RF) magnetron sputtering) to form the homogenous coatings with a stoichiometric composition and control drug release [31,32].

Recently, we have shown that PLLA-based scaffolds can be modified by the plasma, which is formed in the process of DC reactive magnetron sputtering of the titanium target in a dry nitrogen atmosphere [33]. This technique is often called promising for the purposes of the deposition of thin titanium-nitrogen coatings on the surface of thermoplastic polymers with low melting points such as PLLA. It was reported that for the PLLA-based scaffolds, improved hydrophilicity [34] and following biocompatibility [35] are achievable with varying the modification parameters. Moreover, the results were shown to contribute to near-surface effects and not with macro-level changes such as formation of the defects, and not to deteriorate fibrous mats mechanical properties.

The aim of this research is to investigate the influence of the DC reactive magnetron plasma modification on drug release and antibacterial activity of electrospun PLLA scaffolds with incorporated chloramphenicol.

## 2. Materials and Methods

### 2.1. Materials 

Polylactic acid (PLLA, MW = 38 kDa) purchased from (Corbion, Gorinchem, The Netherlands) and hexafluoroisopropanol (HFIP) purchased from (Ekos-1, Moscow, Russia) were used for preparation of the spinning solutions. Chloramphenicol (CHL) powder was purchased from Pharmstandart (Pharmstandart, Moscow, Russia). Phosphate Buffer Saline (PBS, pH = 7.2–7.4) tablets (Biolot, Saint-Petersburg, Russia) were dissolved in distilled water produced by water distiller PE-2205 (A) (ECROSKHIM, Saint-Petersburg, Russia) and used for further drug release modelling.

### 2.2. Preparation of PLLA Fibers by Electrospinning

Two types of polymer solutions were prepared: pure 3 wt./wt.% PLLA/HFIP and chloramphenicol-loaded 50 wt./wt.% chloramphenicol/PLLA in 3 wt./wt.% PLLA/HFIP. The solutions were left for 24 h at room temperature until total dissolution of polymer granules and the drug powder. Total homogenization before electrospinning were obtained by 20 min stirring with a magnetic stirrer at room temperature. Applied electrospinning parameters were: setup—NANON-01A (MECC CO., LTD., Ogori-shi, Japan); directly electrospun solution volume = 8 mL; G26 needle (0.45 mm); spinneret speed = 50 mm/min; flow rate = 3 mL h^−1^; voltage = 20 kV; collector = 200 mm diameter drum type; rotation speed = 50 rpm; syringe-collector distance = 130 mm.

### 2.3. DC Reactive Magnetron Sputtering

To remove the residual solvent, obtained PLLA scaffolds were firstly vacuumated for 10 h at 10−2 Pa. Magnetron sputtering system used for the scaffold surface modification was described earlier by Bolbasov et al. [35]. In this study, the DC mode sputtering of chemically pure titanium (99.99%, Ti) in the dry nitrogen (99.99%, N2) atmosphere was applied with the following parameters: the power discharge = 40 W; the current = 0.2 A; the operating chamber pressure = 0.7 Pa; the magnetron-target distance = 40 mm; the sputtering area = 240 cm^2^; the modification time = 30 s. 

**Figure 1:**
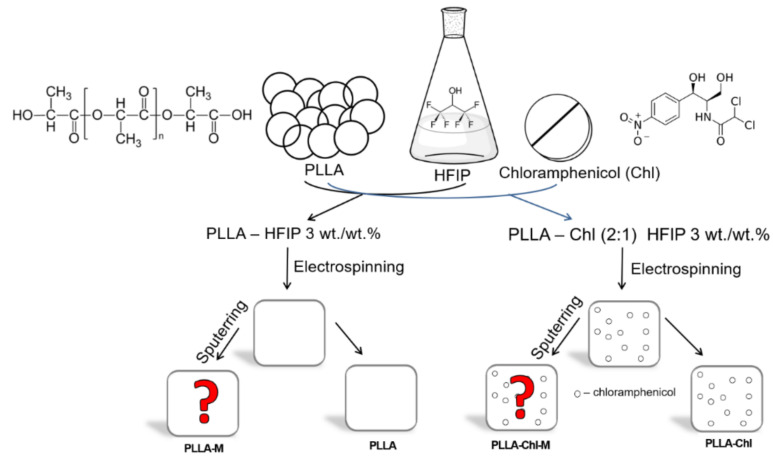
Four groups of experimental samples obtained: PLLA—pure PLLA, PLLA-CHL—chloramphenicol loaded fibers; PLLA-M and PLLA-CHL-M—DC reactive magnetron sputtering modified samples. Squares represent obtained scaffolds. Red question marks represent the samples, which were exposed to the plasma of reactive magnetron sputtering to study its effect on the properties of the materials obtained. “M”—states for “modified”, “CHL”—for chloramphenicol loaded.

All groups of the experimental samples are schematically represented in a Figure 1.

Obtained scaffolds were cut into experimental samples with a surgical scalpel. Average thickness of each scaffold was determined by using an indicator thickness gauge (TN-60, KRIM, Kirov, Russia) with five repetitions for each sample.

The process of the scaffolds’ modification with the plasma of the DC reactive magnetron sputtering was performed as it was described earlier by Bolbasov et al. [35].

### 2.4. Characterization

Scanning electron microscopy (SEM) was applied to investigate changes in the scaffolds’ morphological parameters such as fiber diameter and its quantitative distributions. SEM images were obtained with the scanning electron microscope VEGA3 TESCAN (TESCAN ORSAY HOLDING, a.s., Brno, Czech Republic–France).

Fourier-transform infrared (FTIR) spectroscopy spectra were recorded with Shimadzu XRD6000 spectrometer (Shimadzu Corporation, Kyoto, Japan) to investigate possible changes in chemical structure of both polymer and the drug.

X-ray Diffraction (XRD) was applied to estimate possible changes in polymer crystallinity after the magnetron plasma modification (CuK*α* source, *λ* = 1.54056 Å). Shimadzu XRD 6000 diffractometer (Shimadzu Corporation, Kyoto, Japan) was used. OriginPro 8.1 (OriginLab Corporation, Northampton, MA, USA) and Crystal Impact Match! (Crystal Impact Co, Bonn, Germany) software were used for the diffraction patterns processing.

To investigate possible changes in chemical structure of both polymer and the drug, Fourier-transform infrared spectroscopy (FTIR) spectra were recorded with Shimadzu XRD6000 spectrometer (Shimadzu Corporation, Kyoto, Japan).

X-ray photoelectron spectroscopy (XPS) was implemented to study the surface layers’ elemental composition. A PHI X-tool automated XPS microprobe equipped spectroscope (ULVAC-PHI, Inc, Osaka, Japan) combined with Ar-GCIB and low-energy electron and Ar ions charge neutralizing system were used. The samples were sputtered with Ar for 5 min to remove any residual contamination. A monochromatic X-ray source (AlK*α* source, *λ* = 8.33934 Å) with a 400 µm diameter spot of in size were used for the analysis. The library of the reference XPS spectra and peak deconvolution was provided by Casa XPS software (Casa Software Ltd., Teignmouth, United Kingdom).

The surface wettability of the scaffolds was assessed with water and the Sessile drop method was applied by measuring the static contact angle of water (WCA). Each PLLA scaffold group consisted of three samples with the dimension of 5 × 40 mm^2^. Three droplets with a volume of 3 μL were placed on each sample. The images were captured with Krüss EasyDrop DSA20 contact-angle measurement system (KRÜSS Scientific Instruments, Hamburg, Germany).

The Wenzel roughness value (*r_Wenzel_*) for all samples was calculated based on the following equation [36]:

(1) $$\cos\theta_{W}=r_{Wenzel}\cos\theta_{\Upsilon }$$

where *θ_W_* is the contact angle on a rough surface and *θ_Υ_* is the contact angle on a smooth surface, which is hereby the WCA: for PLLA and PLLA-M samples—of the unmodified pure PLLA film (value of *θ_Υ_* is 98°) and for PLLA-CHL and PLLA-CHL-M samples—of the unmodified film, obtained from chloramphenicol-loaded 50 wt./wt.% chloramphenicol/PLLA in 3 wt./wt.% PLLA/HFIP solution (value of *θ_Υ_* is 103°).

Tensile tests were performed with Instron 3369 testing machine (Illinois Tool Works, Glenview, IL, USA) equipped with 50 N load cell at room temperature. A cross-head separation was used for the strain measurement. Obtained stress–strain curves were processed with Bluehill^®^ Universal software (Illinois Tool Works, Glenview, IL, USA).

For the statistical analysis, Prism (GraphPad Software, San Diego, CA, USA) software and was used. Considering that all the data had non-normal distribution, the Mann–Whitney test was applied. The difference was considered significant at the significance level of *p* < 0.05.

Details on the experimental methodology can be found in the Supplementary Materials (Description S1).

### 2.5. Antibacterial Assay

To study the effect of the samples on Gram-positive and Gram-negative microflora, bacteria *Staphylococcus aureus* subsp. Rosenbach ATCC 6538D-5 and *Escherichia coli* ATCC 25922 (American Type Culture Collection, Masanass, VA, USA) were used as test objects. Antibacterial activity was assessed according to the standard disc-diffusion method, which has been effectively applied to polymeric fibers [13,37].

Sterilization of the samples was carried out by ultraviolet irradiation (*λ* = 265 nm, LEDVANCE UV-C, 55 W, Garching, Germany) of each side in hydrogen peroxide vapor for 40 min in sterile conditions, as this approach is proven to be non-destructive to polymeric materials [38]. UV-light sterilization in chosen conditions does not lead to PLLA degradation (which could possibly result in faster drug release) due to short time of exposition [39]. Sterilization of culture media (1 atm and 120 °C for 30 min) was performed with subsequent pouring into Petri dishes under sterile conditions. Each experimental sample was placed on the surface of a dense agar medium. To simulate conditions similar to the wet environment of a wound, the environment on whose surface the sample was located was changed to a similarly inoculated one every 24 h. In this case, bleeding, outflow of wound discharge, and secondary bacterial infection were modeled.

Antibacterial assay process:

1. Main culture of bacteria was grown primarily. After 24 h, sowing was carried out on solid media to establish the initial number of bacteria and to control the purity of the main culture.
2. For each Petri dish with 15 mL of the corresponding dense nutrient medium, the *S. aureus* or *E. coli* strain was inoculated by the lawn method (0.1 mL of cell suspension at a concentration of (1 × 10^6^) cells/mL) from a pure main culture (Table 1). Then, a square sample was placed in the center of the dish using sterile forceps. Incubation was carried out in a thermostat at a temperature of +37 to 38 °C for 24 h.

3. The duration of the experiment was 7 days for both Gram-negative and Gram-positive microflora. Each group of samples was divided into control and experimental samples. Control samples were kept in the same Petri dish for 7 days. In the case of test samples, they were transferred every 24 h to similarly inoculated Petri dishes, followed by measurement of the inhibition zone.
4. After incubation, the bacterial inhibition zone will be measured with an accuracy of 0.1 mm. The zone of inhibition of growth was first measured 24 h after sowing, then 6 times every 24 h. The larger the zone of bacterial growth inhibition, the higher the antibacterial activity of the sample becomes [40]. The bacterial growth inhibition zones considered the zone of complete growth inhibition for the growth of colonies. Single and very small bacterial colonies in the inhibition zone were ignored.

### 2.6. Drug Release Study

Four 10 × 10 mm^2^ area test pieces were cut from the scaffold mat and weighted to evaluate the predicted amount of the loaded drug. Then, each sample was put into plastic containers filled with 2 mL phosphate-buffered saline (PBS, pH = 7.2–7.4) at 25 °C for 7 days without stirring. At the predetermined time points, a 1 mL aliquot was withdrawn to the test vials and replaced with an identical volume of the fresh medium. The chloramphenicol concentration in the test vials was calculated from the UV–vis spectra analysis: calibration data (dependence of the peak area on the solution concentration) were approximated by linear equation. The spectra were obtained with Shimadzu UV-1280 spectrometer (Shimadzu Corporation, Kyoto, Japan), at a detection wavelength for chloramphenicol of *λ* = 278 nm [41].

### 2.7. Drug Release Modeling

The experimental drug release results and fiber diameter distributions were used during fitting as the input data in the fiber distribution model [42]. Briefly, the model is based on the drug release model from a cylindrical fiber (hereafter referred to as the homogenous model) but it takes into account the observed fiber diameter distribution to more accurately determine an apparent drug diffusion coefficient. Numerical inversions of the Laplace domain solutions and non-linear regressions were carried out using the custom-written program in Python programming language already published in [43].

To clarify the mechanism of drug release from non-modified scaffold, first 60% drug release data were fitted in Korsmeyer–Peppas model [44] (Table S1).

### 2.8. Statistical Analysis

For the statistical analysis, ImageJ 1.44p software (National Institutes of Health, MD, USA) and OriginPro 8.1 software (OriginLab Corporation, Northampton, MA, USA) were used.

## 3. Results and Discussion

### 3.1. Morphology

All obtained spinning solutions were totally homogenized by stirring, the drug powder was fully dissolved forming true solutions. Thus, they performed similar sufficient spinnability and fibrous mats were formed without defects and major thickness fluctuation. It was shown, that adding the chloramphenicol powder to PLLA/HFIP solution does not affect thickness of scaffolds (Table 2). Moreover, further plasma modification did not change this parameter as well.

Electrospun drug-loaded scaffolds implanted in a body act not only as DDS but also as extracellular matrix (ECM). Walles et al. [45] showed that scaffold thickness limits its successful repopulation and revascularization, so we can assume that these processes will not be affected by drug loading and further modification from this viewpoint. Furthermore, drug release depends also on the fiber mat thickness: the thicker the mat—the longer the diffusion path of a drug molecule is, and the longer the swelling and polymer degradation processes take, which results in a more prolonged release [46]. Here we see that plasma modification does not change mat thickness, so all changes in drug release profile are contributed to other factors.

**Figure 2:**
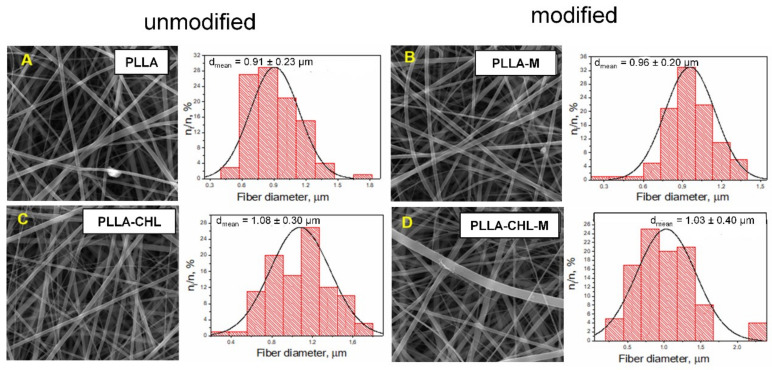
SEM micrographs of obtained electrospun scaffolds: unmodified pure PLLA (**A**), PLLA-chloramphenicol 50 wt./wt.% (**C**) and corresponding DC reactive magnetron plasma modified samples (**B**,**D**), respectively.

Another crucial morphological parameter, which influences drug release process is electrospun fiber diameter. Generally, a drug releases faster from thin fibers rather than from ones with a bigger mean diameter [47,48]. To investigate the effect of magnetron plasma modification on the mean fiber diameter of PLLA and PLLA-CHL scaffolds, SEM images of all groups of samples were analyzed (Figure 2).

Surface of fibers for all samples is mainly smooth with no electrospun-related defects, such as breakages, globules, or diameter fluctuation, even though drug loading is considerably high compared to previous studies [15,16]. The drug crystals were also not observed on the surface of fibers, which is a positive result, since it is known that the formation of an amorphous drug is favored [9].

To investigate the possible changes in the mean fiber diameter after the DC reactive magnetron plasma modification, obtained images were statistically analyzed. Mean fiber diameter for the pure PLLA scaffolds changed from 0.91 ± 0.23 to 0.96 ± 0.20 μm after modification. As for the chloramphenicol-loaded samples, the value of this parameter was in the same interval: 1.08 ± 0.30 μm before and 1.03 ± 0.40 μm after modification, respectively. It can be concluded that both drug loading and plasma modification do not significantly change the mean fiber diameter.

Thus, we can assume that plasma modification with chosen parameters is a non-destructive method because it does not affect the morphology of the obtained scaffolds, and all changes in the drug release profile are, presumably, due to the effects occurring in the surface of each single fiber.

### 3.2. Chemical Composition

Processing electrospun DDS scaffolds can result in several chemical processes such as polymer chain destruction, scission, and covalent bonding between the drug and the polymer matrix. In some cases, these processes are undesirable. If polymer has low molecular weight and degrades too fast, the drug will release faster and scaffold will have poor ECM potentials because of the possible collapse before completed recellularization [45]. Scission may lead to an increase in the polymer molecular weight, and it is known that the drug is more likely to release more slowly from high molecular weight fibers. Covalent bonding makes release almost impossible since it “inactivates” the drug molecule.

So, assessment, regarding whether drug incorporation into the spinning solution and further plasma modification affected the chemical composition of both PLLA and chloramphenicol molecules, is important. We performed FTIR spectroscopy to investigate possible changes in characteristic chemical bonds.

**Figure 3:**
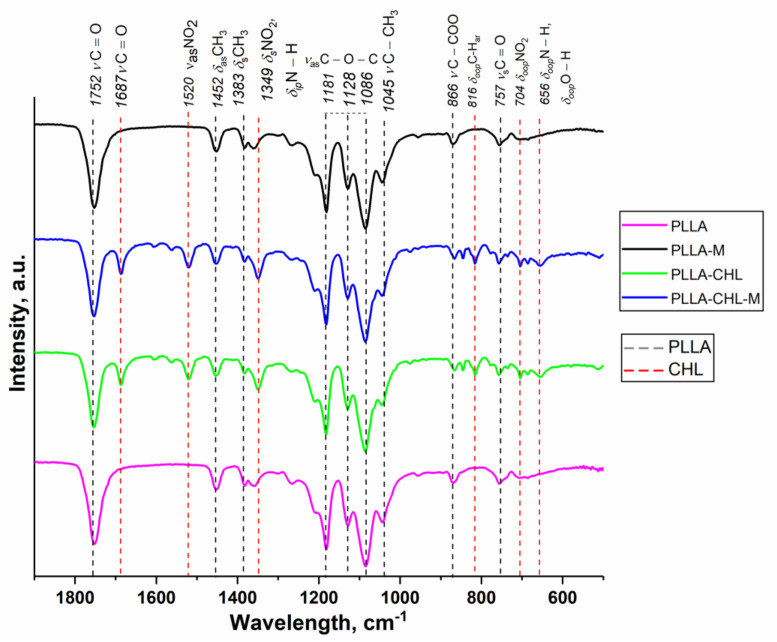
FTIR-spectra of unmodified (pink and green lines) and modified (black and blue lines) electrospun PLLA scaffolds.

For the chloramphenicol-loaded samples, the drug adsorption bands appeared only on FTIR spectra of the drug-loaded materials (Figure 3). Regions 1750–1350 cm^−1^ and 820–560 cm^−1^ on the PLLA-CHL and PLLA-CHL-M samples spectra have respective peaks positioned at the wavelengths similar to ones reported to correspond with chloramphenicol [41] (Table S2).

Characteristic adsorption band of PLLA (1752 cm^−1^, C=O stretching vibrations) did not change its position [49]. Thus, it may be suggested that there were no intermolecular interactions, such as covalent bonding, between PLLA and chloramphenicol during processes of spinning solution preparation and electrospun. Several non-covalent drug–polymer interactions and DDS based on these effects are described in literature [50], but the presence of such interactions cannot be unambiguously established using IR spectroscopy. Magnetron plasma was also shown not to change the position and intensity of both PLLA and chloramphenicol, indicating that the both components did not undergo phase transitions or chemical reactions during the modification process.

**Figure 4:**
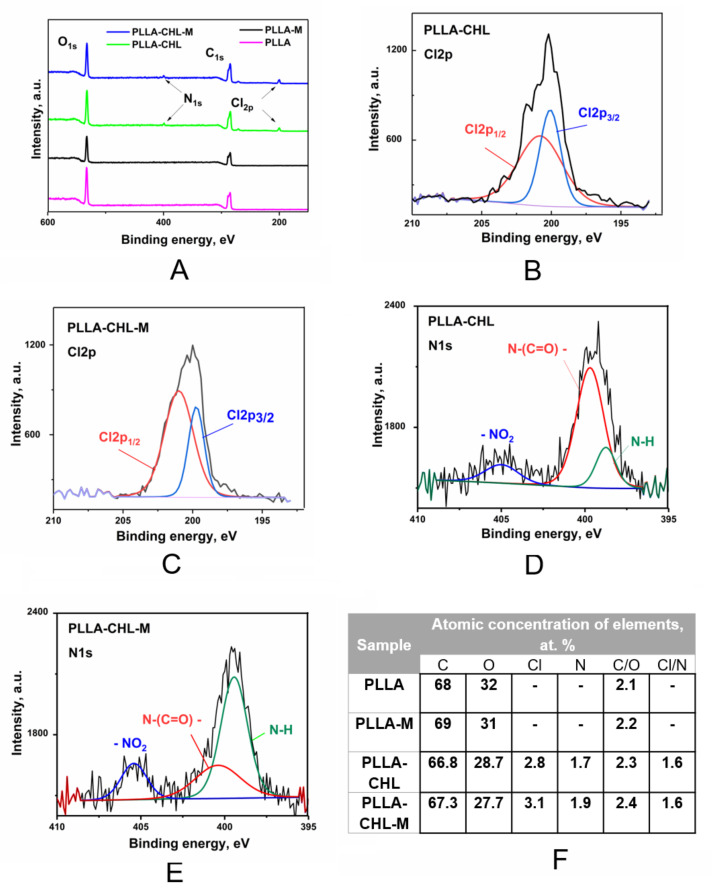
Full XPS survey spectra before and after magnetron plasma modification (**A**) and N1s/Cl2p high resolution core level spectra XPS spectra of samples of the unmodified (**B**,**C**) and modified (**D**,**E**) samples. Estimated atomic concentrations of elements (**F**).

The X-ray photoelectron spectroscopy (XPS) was used to study elemental composition of the obtained PLLA scaffolds’ surface and to study the applied plasma modification effects. It has been demonstrated that the position and shape of the C1s and O1s lines in the control pure PLLA sample spectra corresponded to the reference data on the PLLA bond energies (Figure 4) [51].

On the survey spectra of the chloramphenicol-loaded PLA scaffolds, peaks corresponding to N_1s_ and Cl_1s_ can also be found (Figure 4B–E). These peaks refer to nitrogen and chlorine in chloramphenicol. The peaks were also observed after the etching of the sample surface with argon ions; thus, the inclusion of nitrogen and chlorine verifies the drug incorporation on the PLA surface. According to the data presented in the Figure 4, magnetron plasma modification does not lead to new bonds formation. Thus, we can assume that chemical structure of PLLA surface remains unmodified.

Bolbasov et al. proposed the in-depth empirical model to describe the chemical composition changes in the PLLA scaffold surface as a result of the DC reactive magnetron sputtering modification [52]. It was reported that surface modification process consists of two simultaneous processes: the plasma-induced PLLA destruction, and the formation of the nitrogen-doped TiO_2_-based inorganic thin coating. Moreover, thickness and chemical composition change with an increase in the modification time period. Thus, it is predicted to observe nitrogen and titanium peaks in the N1s and Ti2p XPS spectra of obtained scaffolds. However, there are no Ti2p peaks in the spectra of both modified control and drug-loaded samples. Nitrogen peaks are only observed in the modified drug-loaded sample spectra, and these peaks can be mainly referred to nitrogen in chloramphenicol. This may demonstrate that the process of the PLLA destruction in thin surface layers of scaffolds prevails during the magnetron plasma modification with chosen parameters. A possible reason for this could be the short modification period: 30 s, compared to 2–8 min in [52].

Moreover, redistribution of the chemical bonds content ratio is observed. Comparing the surface modified samples with the unmodified ones, the areas of the O–C=O and –O–CH coordinations of the C1s peak show a slight decrease, and the –CH_3_ increases, which may demonstrate the signs of the polymer destruction (Table 3).

**Figure 5:**
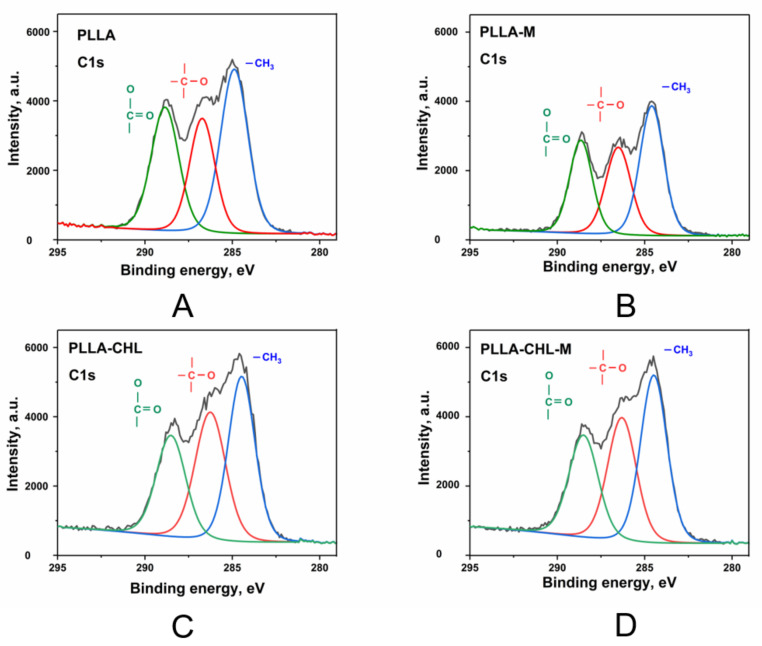
C1s XPS high resolution spectra of the control PLA samples: pure PLLA scaffold (**A**), chloramphenicol-loaded PLLA scaffold (**B**) and magnetron plasma modified samples (**C**,**D**), respectively.

Deconvolution analysis of the high-resolution C1s peak was performed to study the changes in carbon functional groups. The C1s core-level spectra with the curve-fittings for –CH_3_ (~285 eV), C–O (~287 eV), and (O–C=O) (~289 eV) bonds of the control, unmodified, and plasma-modified PLA scaffolds are shown in a Figure 5.

In the drug-loaded samples, C1s XPS spectra peaks in 284–287 eV are observed, which corresponds to C-N bonds in chloramphenicol. These changes are not dramatic, confirming the fact that during 30 s of plasma modification only “loosens” the surface of polymer scaffolds.

Morent et al., in their work, listed the possible main factors resulting in the surface of the PLLA scaffolds degradation induced by balanced magnetron plasma [53]. They are: the energy of neutralized ions reflected from the target, the kinetic energy of the deposited atoms and plasma irradiation, and the condensation energy of the sputtered atoms and ions energy. The proposed process is defined by formation of functional nitrogen-containing groups as a product of reaction between formed polymer radicals and N radicals. Moreover, authors underline that reactions between two polymer radicals are less preferable than ones with N radicals. Thus, a PLLA surface with a low degree of crosslinking is formed. We propose that 30 s of modification time is sufficient for polymer radicals’ formation, but not for absorbing enough nitrogen atoms from the atmosphere to form the amount of bonds above the XPS detection level.

### 3.3. Study of Polymer Crystallinity

The incorporated drug release profile is known to be dependent on the degree of the scaffold material crystallinity. Highly dense crystalline regions are less likely to release incorporated small drug molecules through the obstructed diffusion of drug molecules, than amorphous ones [54,55,56]. It was also reported that incorporating the drug intro polymer matrices can change polymer crystallinity, and this effect is dependent on drug/polymer ratio [57,58,59]. To estimate the effect of the chloramphenicol presence in the PLLA scaffold, and further DC reactive magnetron modification on the polymer crystallinity, XRD analysis was performed.

**Figure 6:**
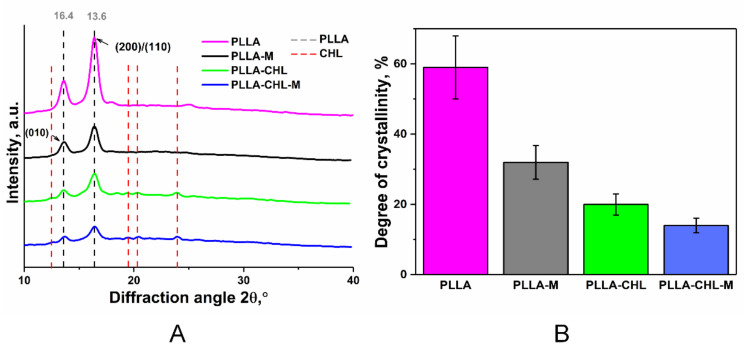
XRD spectra: (**A**) of PLLA scaffolds before DC reactive magnetron modification (pink and green lines) and after DC reactive magnetron modification (black and blue lines). (**B**) Degree of crystallinity of PLLA scaffolds.

XRD patterns of the pure PLLA and PLLA/chloramphenicol 50 wt./wt.% scaffolds are shown in a Figure 6A.

Characteristic peaks of the PLLA (*2θ* = 14° and 16°) can be observed on the XRD-pattern contributed to the unmodified pure PLLA scaffold [60]. The diffraction patterns of pure PLLA scaffolds show differences from that of drug loaded ones. Chloramphenicol characteristic peaks can be observed with *2θ* at 13° and around 20° [37]. The absence of several expected peaks, such as in region *2θ* = 30°, can be explained by the fact that raw drug crystallization in polymeric solutions is limited [61]. So, we can assume that chloramphenicol exists in a less crystalline state in scaffolds than in raw powder. The effect of adding the drug on the polymer crystallization process is also clearly seen in Figure 5. Chloramphenicol-loaded samples are less ordered than those without drugs. This result may be attributed to the competition in the crystallization process of chloramphenicol and PLLA during the electrospun [62].

The degree of scaffold crystallinity decreases after DC reactive magnetron modification (Figure 5B). These changes could be attributed to the reorientation of the shorter chains of polymer during short term thermal exposition. Average crystallite size before and after modification was also calculated: 11.8 ± 0.1 nm and 11.7 ± 0.3 nm for pure PLLA samples; 13.6 ± 3.8 nm and 11.7 ± 3.4 nm for chloramphenicol-loaded samples. The results show that crystallite size does not differ within the margin of error, which indicates that polymer crystallization is more likely associated with the increase in the number of crystallites, rather than with their growth.

### 3.4. Wettability

**Figure 7:**
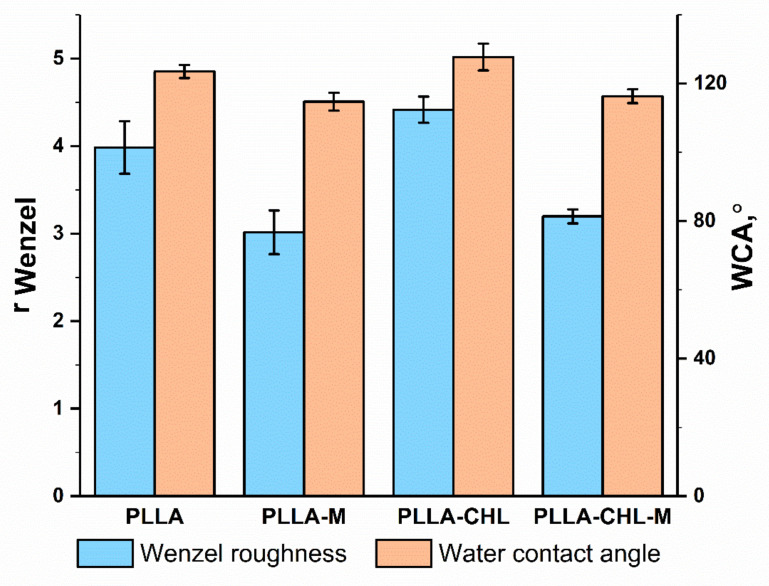
Water contact angle (WCA) and Wenzel roughness value of the electrospun PLLA scaffolds depending on the modification with the DC reactive magnetron plasma.

Another crucial characteristic of scaffolds as DDS and ECM-mimicking materials is the wettability of their surface with water, as water is the basis of most biological fluids. It is important not only for successful integration with the body, but also for the release of the incorporated drugs [47]. The drug release rate is higher if wetting and swelling processes are facilitated [63]. Therefore, the water contact angle was chosen as a parameter for investigation whether or not the chloramphenicol incorporation loading and DC reactive magnetron affect the scaffolds wettability (Figure 7).

The surface of all studied samples was hydrophobic, which is expected, since PLLA is a synthetic polymer, but there is a tendency to increasing the wettability. Similar results were also described in [34]. Maryin et al. in [64] reported the increase in wettability of PLLA scaffolds obtained from 5, 9, and 14 wt./wt.% solutions of the in trichloromethane after DC reactive magnetron sputtering modification for 2, 4, and 8 min. It was show that scaffolds obtained from higher PLLA content solutions are more susceptible to a decrease in contact angle. Moreover, longer modification was characterized by a more pronounced effect. Considering the fact that in this work we used only a 3 wt./wt.% solution and processed the scaffolds for 30 s, the results obtained can be considered consistent with the positions put forward by the authors.

The surface roughness is another important parameter affecting on wettability [65]. In the case of a rough surface, the drop on the surface does not have an ideal shape, but is deformed under the action of the force of gravity. The liquid ”flows” into the grooves and pores, which leads to the formation of a contact angle, the value of which is different from that on a smooth surface of the same chemical composition. If the liquid completely fills the grooves and pores, forming a liquid–solid interface, then this case is called the Wenzel condition. However, if the liquid does not completely flow into the grooves and pores, and air bubbles remain in them, then two interphase boundaries are formed: liquid–solid and liquid–gas. This case is called the Cassie–Baxter condition. To many materials (non-superhydrophobic), the Cassie–Baxter condition is not a stable and energetically favorable one: pressure or shaking can easily lead to replacement of air in pores by liquid [66]. In this paper, we use the assumption that water droplets on the surface are in full contact with the scaffold surface. As well as liquid media during drug release, constant stirring before and during sampling makes the presence of air in the space between the fibers an unlikely occurrence.

It is known that at equal values of the average fiber diameter, the surface of scaffolds with a higher roughness demonstrates a lower wettability [67]. The impact of roughness on the contact angle can be calculated by the Wenzel equation, using WCA on a rough surface (here—scaffolds) and on a smooth surface (here—film). Two types of films were prepared by airdrying the 3 mL of PLLA/HFIP and PLLA/CHL/HFIP solutions on a glass surface with further vaccuumating for removing the residual solvent. WCA was measured by sessile drop method. It was shown that the contact angle of drug-loaded samples is higher compared to pure polymer film: $\theta_{\Upsilon }\;$= 103 ± 3° for PLLA-CHL film against $\theta_{\Upsilon }\;$= 98 ± 2° for pure PLLA film. This can be justified given the poor water solubility of chloramphenicol. In Figure 7, the Wenzel roughness for unmodified PLLA and PLLA-CHL scaffolds are: *r_Wenzel_* = 3.98 ± 0.30 and *r_Wenzel_* = 2.72 ± 0.16, respectively. After the DC reactive magnetron plasma modification, the Wenzel roughness decreases for both samples to: *r_Wenzel_* = 3.01 ± 0.25 and *r_Wenzel_* = 1.97 ± 0.08, respectively. This can potentially explain the better wettability of the modified drug-loaded sample.

### 3.5. Drug Release Study

The release of incorporated drugs from fibers is a complex process that includes several parallel processes: desorption of the surface-localized drug, drug molecules’ diffusion from the fiber bulk to the release medium, and degradation of the polymer matrix followed by release of entrapped drug, especially from crystalline parts. The graphs below show the results for 1 day of the experiment, because all curves reached a plateau during this period.

**Figure 8:**
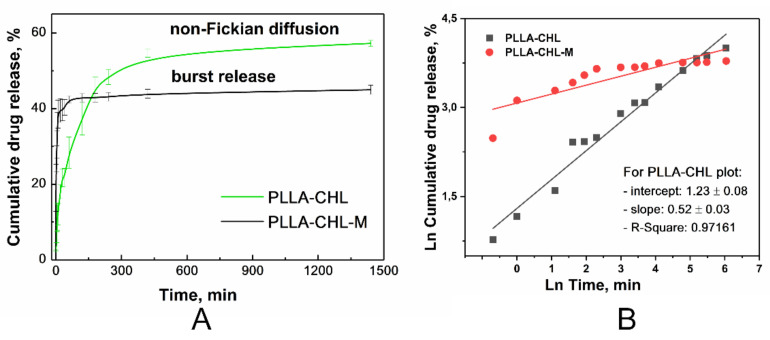
Chloramphenicol release kinetics profile from monolayer electrospun PLLA scaffolds: (**A**) Experimental curves for samples before (black line) and after (green line) DC reactive magnetron modification. (**B**) Results of the Korsmeyer–Peppas approximation: before (black squares) and after (red circles) magnetron modification.

Chloramphenicol release experimental curves from monolayer materials are shown in a Figure 8A.

As suggested by us before [68], TTP (time to plateau) and QTP (quantity to plateau) values can be determined for each kinetic curve. The TTP value decreased from 1440 to 60 min, but the QTP value has shown a less drastic change—from 57.6 ± 0.8 wt./wt.% to 44.9 ± 1.2 wt./wt.%.

The burst release is characteristic for both kinetic curves, but release profiles vary. Release from PLLA-CHL-M (modified) can be classified as “immediate” with a very low TTP. Such results could be hard to obtain for drugs with limited aqueous solubility. Some techniques that result in obtaining ultrafast release require changing the electrospun setup: shifting from direct current to alternate current [69].

Chloramphenicol release from an unmodified antibiotic loaded sample can be primarily classified as anomalous (non-Fickian) diffusion because the diffusion exponent obtained from data fitting using the empirical Korsmeyer–Peppas equation [44] was 0.49 (Figure 8B). The data obtained for the drug release from modified scaffolds fitting cannot be analyzed because they do not meet the principle of Korsmeyer–Peppas model (*R^2^* = 0.71538).

Magnetron processing of materials decreases TTP. Therefore, most of the drug that could transport to release medium via desorption and thin layer diffusion is released in the first hour of the experiment. QTP is also affected but to a lesser extent. In other words, magnetron modification allows for the obtaining of a high drug concentration in the short term, but overall, the release rate decreases.

Despite the ability of polylactide to degrade in the release medium, the degradation impact on drug release in the early time periods is negligible. Hence, we hypothesized that the release is initially attributed only to a diffusion of the drug from the fibers into the medium. Korsmeyer–Peppas model does not consider fibrous structure of the scaffold. Therefore, the diffusion coefficient of chloramphenicol was determined based on the fitting of the experimental data and on fiber diameter distribution.

Table 3 shows the differences in scaffold mean fiber diameter (*d_mean_*) and coefficient of variation, which was calculated based on the following Equation [43]:

(2) $$CV=\frac{s_{N}}{R_{mean}}$$

where *S_N_* is a standard deviation of a discrete random variable, and *R_mean_* is a mean fiber radius before and after plasma modification.

**Figure 9:**
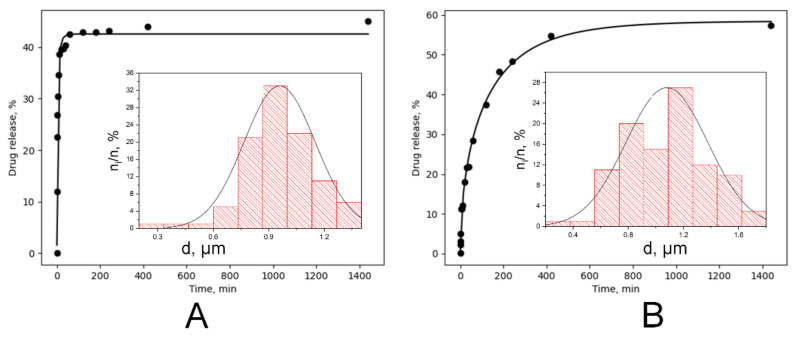
Simulation results of the release kinetics of chloramphenicol: Results of chloramphenicol release kinetics program simulation: monolayer electrospun PLLA scaffolds before (**A**) and after (**B**) DC reactive magnetron modification. Inset diagrams present the fiber diameter distributions of the corresponding scaffolds.

Because of the experimental data fitting (Figure 9) with the fiber distribution model, the apparent diffusion coefficient of the drug was found to be *D* = 2.42 × 10^−13^ cm^2^ s^−1^ for the case of chloramphenicol release from the unmodified PLA samples.

The values of the apparent drug diffusion coefficient, which were obtained after the fitting of the release curves of unmodified and magnetron modified samples are presented in Table 4. This result confirms that the release mechanism of chloramphenicol from the PLA scaffold was modified during the magnetron sputtering modification. The amount of instantly released drug, Q_0_, was used as an additional fitting parameter (Table 3), leading to a significant improvement of regression quality. Before the irradiation, the ability of the chloramphenicol to be eluted is restricted by a hydrophobic polymer surface. In contrast, the magnetron sputtering modification allowed for an instant availability of approximately 25% of the drug. This is most likely the amount of the drug located on the surface of the electrospun fibers.

In the example of a chosen model drug, increased surface wettability of the scaffolds after the modification with magnetron sputtering was demonstrated to allow for an increased delivered drug dosage within a short time period. Moreover, based on the results of the mathematical modeling, the drug diffusion coefficient was changed during the magnetron modification. Thus, the drug release mechanism was altered presumably due to induced decrease in scaffolds crystallinity followed by relaxation of the internal stresses as a result of a short-term thermal exposure and increase in surface wettability. All listed factors are potent to facilitate the diffusion of the drug molecules.

As we mentioned before, burst release is most often described by researchers as an undesirable outcome in the development of drug delivery materials. However, in the considered system (incorporated antibiotic) burst release is a desirable profile, as it helps to achieve the minimum inhibitory concentration and suppress/control already existing infection [70,71]. In a sustained release profile, all the time during which the concentration of drugs, such as antibiotics or chemotherapeutical agents [72], increase in growth up to desired effective level, is a “wasted” time, as they are ineffective below certain concentration. Here, we show that the use of modifying the surface of a polymer carrier with a DC magnetron plasma makes it possible to obtain a release profile that is more appropriate for the tasks of the antibiotic—chloramphenicol.

### 3.6. Mechanical Testing

PLLA, and electrospun scaffolds based on it, has demonstrated to be a promising material for tissue engineering in a variety of studies [35,73,74]. It should be noted that the role of a drug carrier can be combined with tissue engineering applications when the mechanical properties of materials and their surface morphology do not undergo significant changes when a pharmacologically active substance is added to the composition [75]. Thus, despite the confirmed non-destructive behavior of the DC reactive magnetron modification to the morphology of nanofibers and its potential to control the drug release, it is important to assess it as if it does not deteriorate the mechanical properties of the obtained materials. Among other mechanical stresses, tensile stress is key to assess the ability of scaffolds to maintain structural integrity during insertion/implantation, tissue repair/cell growth, and under the influence of physiological activities [76].

**Figure 10:**
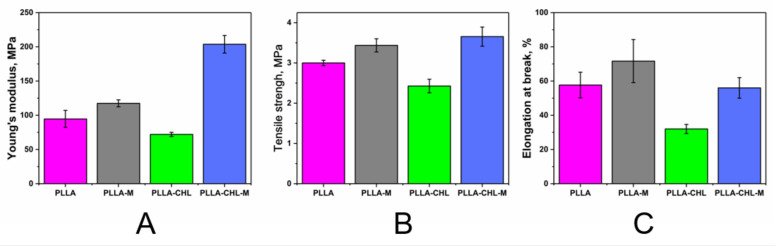
Tensile testing results for electrospun PLLA scaffolds before DC reactive magnetron modification (pink and green bars) and after DC reactive magnetron modification (grey and blue bars): Young’s modulus (**A**), Tensile strength (**B**), and Elongation at break (**C**) were calculated from stress strain curves for *n* = 5 samples of each.

Results of tensile properties testing are presented in a Figure 10.

The chloramphenicol presence in the spinning solution results in changes in the mechanical properties of the resulting fibrous mat. Drug-loaded samples show lower values of the elongation at break and tensile strength than pure PLLA scaffolds. Young’s modulus also decreases with chloramphenicol loading. Presumably, the reason for this phenomenon may be the presence in the fiber structure of areas with an increased concentration of chloramphenicol, which act as stress concentrators. Chloramphenicol is readily soluble in HFIP; therefore, nonuniform distribution of the drug along the fiber is likely during the shock evaporation of the solvent, which accompanies the electrospun process.

DC reactive magnetron modification has an effect on the mechanical properties of both modified and unmodified groups of samples. It increases both their elongation at the break, Young’s modulus, and maximum tensile strength. It is important to mention that the effect on the drug-loaded samples is more prominent. This behavior may be attributed to decreased material crystallinity because mean fiber diameters and distribution curves show no significant change. Polymer chains in an amorphous region are more flexible, so it is easier for material to reversibly stretch and rearrange their supramolecular structure before a total break.

It can be concluded that the plasma of DC reactive magnetron modification has a positive effect on the scaffolds’ mechanical properties. All parameters’ values corresponding to modified chloramphenicol-loaded samples are higher than ones characterizing pure PLLA scaffolds, and it is known that pure PLLA scaffolds were shown to successfully perform as DDS and ECM materials. So, magnetron-plasma modified PLLA/chloramphenicol samples are suitable to further medical application from the perspective of mechanics.

### 3.7. Antibacterial Activity

In this research, unloaded PLLA and PLLA-M samples did not present a significant antibacterial effect: no bacterial growth inhibition zones were observed in test dishes during experiment, meaning that PLLA has no antibacterial activity. These results correspond well to the literature—a weak antimicrobial activity of PLLA and PLLA samples against *S. aureus* and *E. coli* have already been reported [77]. It was also shown that magnetron sputtering of the titanium target in the proposed regiment does not provide the scaffolds’ surface with antibacterial properties. This, presumably, may be due to short modification time, because it is known that longer magnetron modification can lead to Ti-based bacteriostatic structures [78].

Two different models were applied to study the antibacterial activity of the scaffolds obtained: the conventional disc-diffusion method and the “infiltration model”. In both models, a dense agar medium was used as a surface for the samples. No extraneous microflora was found on the nutrient medium: all lawn and colonies were morphologically assigned to the test object used. Chloramphenicol diffused from the scaffolds into the medium forming a bacterial growth inhibition zone.

**Figure 11:**
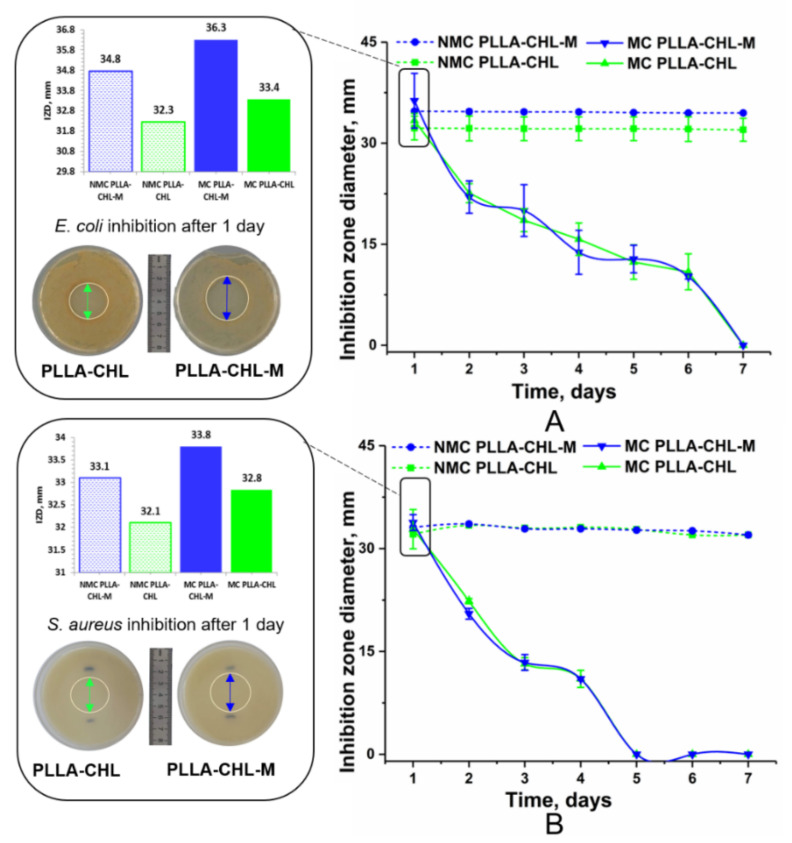
The dynamics of the inhibition zone diameters (IZD) during the experiment: conventional disc-diffusion method “NMC”—dashed lines, infiltration model “MC”—solid lines, unmodified samples—green lines, modified samples with magnetron sputtering—blue lines; (**A**)—Gram-negative microflora (*Escherichia coli*); (**B**)—Gram-positive microflora (*Staphylococcus aureus*).

To simulate the antibacterial activity of the samples under conditions of skin damage, the test samples were transferred to similarly inoculated Petri dishes every 24 h, followed by measuring the zone of inhibition. In this case, such parameters as body fluid infiltration, bleeding, outflow of exudate, and secondary bacterial infection were modelled. Samples without a change in the culture medium were called “NMC” (“no medium change”) samples, and ones with a change in the culture medium were called “MC” (“medium change”) samples. Results of inhibition zones diameter measurements for both models are shown in a Figure 11 (conventional disc-diffusion method “NMC” is presented by dashed lines, infiltration model “MC” is presented by solid lines).

As a result of the bacteriostatic testing, it was found that the samples of the PLLA-CHL and PLLA-CHL-M groups showed significant antibacterial activity. This may be attributed to the activity of chloramphenicol, because it is known that the test objects used are highly sensitive to this drug [79], and we confirmed that PLLA matrices are inert. The antibacterial activity of the samples in both NMC and MC models for the unmodified and modified groups of samples against both Gram-positive and Gram-negative microflora did not differ after the first day of the experiment (*p* > 0.05). However, the activity of PLLA-CHL-M group on the first day is slightly higher than that of PLLA-CHL group (Figure 11). These results correlate with drug release experiments: magnetron sputtering increases the drug release rate in the first day of exposition.

The observed activity of scaffolds in different models has a different character: without a change in the medium, the inhibition zones are constant, and with a change in the medium, their diameter decreases during the experiment. In relation to Gram-negative microflora, activity lasts longer than in relation to Gram-positive ones. Time periods where samples perform antibacterial activity against Gram-negative (6 days) and Gram-positive microflora (4 days).

This is expressed as a statistically significant decrease in the diameter of the suppression zones of microflora growth with each change in the medium (*p* < 0.05). As seen from the graphs, the antibacterial activity of the samples against the Gram-negative microflora decreases gradually. At the same time, the decrease rate in the level of activity is close to linear (Figure 11A). In the case of Gram-positive microflora, the decrease rate in the activity level in the first 4 days is also close to linear with a greater absolute slope than one for the Gram-negative microflora (Figure 11B). The decrease in the antibacterial activity of scaffolds is attributed to the fact that the drug is constantly released from the polymeric matrices into the changing medium, as it occurs in the body with the body fluids infiltration. Thus, the results show that PLLA-CHL and PLLA-CHL-M scaffolds could successfully prevent bacterial growth.

## 4. Conclusions

In this study we obtained the poly (lactic acid)/chloramphenicol scaffolds with high drug loading (50 wt./wt.%) and modified them in plasma of direct current reactive magnetron sputtering of a Ti target. It has been shown that DC reactive magnetron plasma modification leads to burst drug release from the fibers. The main reasons for the effects observed are the decrease in polymer crystallinity, and the increase in wettability accelerating swelling and further diffusion. To confirm the formulated theses, an additional thorough study of the surface of materials using atomic force microscopy (AFM) and dynamic wettability is proposed.

Advanced mathematical modelling of the drug release process was applied to access the changes in diffusion coefficient after magnetron processing. It was shown that the value of the diffusion coefficient changes by two orders of magnitude, which corresponds well with experimental data. We demonstrated that delivery of chloramphenicol using magnetron plasma modification for scaffolds’ surface modification can be used as a tool for a more effective inhibition of both *S. aureus* and *E. coli* growth in the short term, while not reducing the overall antibacterial activity of the drug-loaded samples.

The developed approach can be effective for expanding the possibilities of using polymer scaffolds. Mechanical properties and morphological parameters do not deteriorate as a result of the proposed modification, which means that scaffolds can still be used for tissue-engineering applications. In addition, the presence of an antibacterial agent in their structure makes them active participants in the regeneration process, especially at the initial stages of implantation, when the chance of developing surgical site infections is relatively high. Moreover, a local use of the “outdated” antibiotics may not only prevent the occurrence of some dangerous side effects such as biofilm formation, but also work with the challenging issue of antibiotic resistance. It is known that for many patients who have developed resistance to modern drugs, the use of drugs such as chloramphenicol could be a solution, but it is the side effects and the overall negative effect on the body with systemic use that make this often impossible. It should also be noted that the proposed combination of the introduction of a drug substance into fibers through the stage of a spinning solution followed by modification of the scaffold with a DC reactive magnetron discharge plasma can be used not only for antibiotics, but also for a wide range of drugs. The range includes cytostatics, applied at the tissue resection site for the prevention of the recurrence of cancer, and in other areas where rapid achievement of high drug concentrations is necessary to reach a therapeutic effect.

## Figures and Tables

**Table 1:** Nutritional medium composition.

Ingredients	*Escherichia coli*		*Staphylococcus aureus*	
	Solid Medium	Liquid Medium	Solid Medium	Liquid Medium
Casein tryptone, g L^−1^	10	10	-	-
Yeast extract, g L^−1^	5	5	-	-
NaCl, g L^−1^	10	10	4	4
Fishmeal hydrolysate, g L^−1^	-	-	8	8
Meat peptone, g L^−1^	-	-	8	8
Egg yolk solution, mL	-	-	200	-
Bacteriological agar, vol.%	1.5–2	-	1.5–2	-

**Table 2:** Thickness of unmodified and modified PLLA scaffolds.

Samples	Sample Thickness, µm	
	Unmodified	Modified
PLLA	128 ± 20	128 ± 21
PLLA-CHL	110 ± 20	112 ± 20
PLLA-M	118 ± 30	117 ± 18
PLLA-CHL-M	112 ± 24	112 ± 20

**Table 3:** Results of the PLLA C1s peak deconvolution [51].

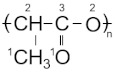	Content of Bonds in C1s (at. %)		
	1 –CH_3_	2 C–O	3 (O–C=O)
Sample	Binding energy (eV)		
	285.00	286.98	289.06
PLLA	41.7	31.5	26.8
PLLA-M	41.8	29.7	28.5
PLLA-CHL	39	34.6	26.4
PLLA-CHL-M	40.5	32.2	25

**Table 4:** Mean fiber diameter (*d_mean_*), coefficient of variation (*CV*), and diffusion coefficient of the drug (*D*) modeling results.

Sample	*d_mean_*, µm	*CV*	*Q_0_*, %	*D* (×10^−13^), cm^2^ s^−1^
PLLA-CHL	1.08	0.30	1.00	2.42
PLLA-CHL-M	1.03	0.40	25.00	106.25

## Supplementary Materials

The following are available online at https://www.mdpi.com/article/10.3390/polym14030373/s1, Description S1: Experimental methodology details, Table S1: Release exponent and a corresponding diffusion mechanism (cylindrical shape), Table S2: Observed adsorption bands of chloramphenicol.
