# Supplementary material for: Reactive Magnetron Plasma Modification of Electrospun PLLA Scaffolds with Incorporated Chloramphenicol for Controlled Drug Release
Source: Polymers (Basel). 2022 Jan 18;14(3):373. doi: 10.3390/polym14030373 (PMC8839200; doi:10.3390/polym14030373)
Supplement: Supplementary file 1 [file polymers-14-00373-s001.zip › polymers-1549814-supplementary.pdf]

Supplementary information

Description S1: A. Experimental methodology details

1. Scanning electron microscopy (SEM)

SEM was applied to investigate changes in the scaffolds' morphological parameters such as fiber diameter and its quantitative distributions. To avoid the negative charge accumulation on the dielectric surface of polymeric scaffold, a 5 nm gold film was sprayed onto the samples fixed on the metallic stud with the double-sided conductive tape. SEM images were obtained with the scanning electron microscope VEGA3 TESCAN (TESCAN ORSAY HOLDING, a.s., Brno, Czech Republic – France). To evaluate fiber diameter distributions, images were analyzed with ImageJ 1.44p software (National Institutes of Health, MD, USA) and OriginPro 8.1 software (OriginLab Corporation, Northampton, USA). To fulfill statistical purposes, three images were taken from each sample and one hundred fibers were randomly selected.

2. Fourier-transform infrared spectroscopy

To investigate possible changes in chemical structure of both polymer and the drug, Fourier-transform infrared spectroscopy (FTIR) spectra were recorded with Shimadzu XRD6000 spectrometer (Shimadzu Corporation, Kyoto, Japan). Wavelength range applied – 550 to 2300 cm^{-1}. spectra processing software – OriginPro 8.1 (OriginLab Corporation, Northampton, USA).

3. X-ray Diffraction (XRD)

XRD was applied to estimate possible changes in polymer crystallinity after the magnetron plasma treatment. Shimadzu XRD 6000 diffractometer (Shimadzu Corporation, Kyoto, Japan) was used with the following parameters set up: 2θ within 10 to 40° range (0.02° step); signal collection time – 2 s. CuKα source; accelerating voltage – 40 kV.

To calculate the crystallite sizes, the Scherrer equation was applied [1]:

$$L_{hkl} = \frac{K\lambda}{\beta \cos\theta}, \tag{1}$$

where L_{hkl} is size of the ordered (crystalline) domains; β is the full width at half maximum; θ is the scattering angle of the PLLA main peaks (2θ = 14° and 2θ = 16°); λ is the X-ray wavelength (here $\lambda = 1.54056$ Å); K is constant (here K = 0.94).

Degree of crystallinity was calculated as a percentage of the scattered intensity of the crystalline phase over the scattered intensity of the crystalline and amorphous phases [2]:

$$\chi = \frac{\sum_{i=1}^{n} S^{i}_{cryst}}{S_{amorph} + \sum_{i=1}^{n} S^{i}_{cryst}}, \tag{2}$$

where S^{i}_{cryst} is the crystalline peak area; S_{amorph} is crystalline halo area.

For the diffraction patterns processing, OriginPro 8.1 (OriginLab Corporation, Northampton, USA) and Crystal Impact Match! (Crystal Impact Co, Bonn, Germany) software were used.

4. Wettability

To assess the scaffolds' surface wettability with water the Sessile drop method was applied. Five water droplets (each 30 µL) were deposited at different spots on the surface of the respective sample and after one minute the images were captured with a Krüss EasyDrop contact-angle measurement system (KRÜSS Scientific Instruments, Hamburg, Germany). The mean value and a standard deviation were calculated. All experiments were carried out in triplicates.

5. Mechanical properties

Tensile tests were performed with Instron 3369 testing machine (Illinois Tool Works, Glenview, USA) equipped with 50 N load cell at room temperature. For each group of samples, five test pieces with 60×20 mm area were cut and fixed in the machine so that the initial length was set at 10 mm. Then a constant load was applied with a cross-head speed set at 10 mm/min. A cross-head separation was used for the strain measurement. Obtained stress-strain curves were processed with Bluehill® Universal software (Illinois Tool Works, Glenview, USA).

6. Drug release modeling

The experimental drug release results and fiber diameter distributions were used during fitting as the input data in the fiber distribution model [3]. Briefly, the model is based on the drug release model from a cylindrical fiber (hereafter referred to as the homogenous model) [4], but it takes into account the observed fiber diameter distribution to more accurately determine an apparent drug diffusion coefficient. For each type of sample under analysis, mean fiber diameter was calculated. The total released drug amount, which takes into account the fiber distribution, was determined as follows:

$$\hat{Q}_{total}(s) = \frac{Q_0}{s} + M_{total} \sum_{i=1}^{n} \frac{R_i^2}{\overline{R}^2} p(R_i) \frac{2}{s\sqrt{st_{di}}} \frac{I_1\left(\sqrt{st_{di}}\right)}{I_0\left(\sqrt{st_{di}}\right)} , \qquad (3)$$

where $\hat{Q}_{total}(s)$ is the Laplace transform of the amount of a drug released from the fibers, Q_0 is the amount of immediately released drug, M_{total} is the total amount of a drug loaded into the fibers, R_i is the radius of i-th group of fibers (i is the column number in the fiber distribution), and $p(R_i)$ is the probability density of an i-th fiber group (the height of i-th column in the fiber distribution). Further, $\overline{R}^2 = \sum_{i=1}^{n} R_i^2 p(R_i)$, $t_{di} = \frac{R_i^2}{D}$ is the characteristic time of diffusion, I_0 and I_1 are the modified Bessel functions of the first kind, zero and first order, respectively. Numerical inversions of the Laplace domain solutions and non-linear regressions were carried out using the custom-written program in *Python* programming language already published in [5]. An equal weighting was used for nonlinear regressions.

To clarify the mechanism of drug release from non-treated scaffold, first 60% drug release data were fitted in Korsmeyer-Peppas model [6].

$$Mt/M_\infty = K \times t^n \ , \tag{3}$$

where M_t/M_∞ is a fraction of drug released at time *t, K* is the release rate constant, and *n* is the release exponent. The *n* value is commonly used to divide different release mechanisms for cylindrical shaped matrices, such as cylindrical tablets. To get the *n* value, the data obtained from the drug release study (see 2.8) were recalculated and plotted as log cumulative drug release vs log time. Data were approximated by linear equation and a slope value was taken as the release exponent. The boundary values of this parameter and the mechanisms corresponding to the ranges are presented in Table S1.

Table S1. Release exponent and a corresponding diffusion mechanism (cylindrical shape) [7,8]

Release exponent (n)	Diffusion mechanism
$0.45 \leq n$	Fickian diffusion
$0.45 < n < 0.89$	non-Fickian diffusion
$n = 0.89$	Case II (relaxational) transport
$n > 0.89$	Super Case II transport

B. FTIR study

Table S2. Observed adsorption bands of chloramphenicol [9,10]

Adsorption band, cm^{-1}	Corresponding vibration
1752	C=O
1687	C=O stretch
1520	NO_2 asym. stretch
1349	δ_s NO_2, δ_{ip} NH
1181	
1128	ν_{as} C–O–C
1086	
816	C–H_{ar}
704	NO_2 wagging
565	O–H o.p., C N–H o.p.

References

1. Kawai, F.; Nakadai, K.; Nishioka, E.; Nakajima, H.; Ohara, H.; Masaki, K.; Iefuji, H. Different enantioselectivity of two types of poly(lactic acid) depolymerases toward poly(l-lactic acid) and poly(d-lactic acid). *Polym. Degrad. Stab.* **2011**, doi:10.1016/j.polymdegradstab.2011.03.022.
2. Mo, Z.; Zhang, H. The Degree of Crystallinity in Polymers by Wide-Angle X-Ray Diffraction (Waxd). *J. Macromol. Sci. Part C* **1995**, *35*, 555–580, doi:10.1080/15321799508021751.
3. Petlin, D.G.; Amarah, A.A.; Tverdokhlebov, S.I.; Anissimov, Y.G. A fiber distribution model for predicting drug release rates. *J. Control. Release* **2017**, doi:10.1016/j.jconrel.2017.05.021.
4. Zhuravlev, M.V.; Remnev, G.E.; Shubin, B.G. Volume Self–Sustained Discharge in Atmospheric Pressure Gas with High Pulse Repetition Frequency. *Appl. Mech. Mater.* **2015**, doi:10.4028/www.scientific.net/amm.756.269.
5. Spiridonova, T.I.; Tverdokhlebov, S.I.; Anissimov, Y.G. Investigation of the Size Distribution for Diffusion-Controlled Drug Release From Drug Delivery Systems of Various Geometries. *J. Pharm. Sci.* **2019**, *108(8)*, 2690–2697, doi:10.1016/j.xphs.2019.03.036.
6. Singhvi, G.; Singh, M. In-vitro drug release characterization models. *Int J Pharm Stud Res* **2011**.
7. Peppas, N.A. Controlled release: A quantitative treatment. *J. Control. Release* **1990**, doi:10.1016/0168-3659(90)90169-t.
8. Siepmann, J.; Peppas, N.A. Modeling of drug release from delivery systems based on hydroxypropyl methylcellulose (HPMC). *Adv. Drug Deliv. Rev.* 2001.
9. Kumar Trivedi, M. Spectroscopic Characterization of Chloramphenicol and Tetracycline: An Impact of Biofield Treatment. *Pharm. Anal. Acta* **2015**, doi:10.4172/2153-2435.1000395.
10. Sajan, D.; Sockalingum, G.D.; Manfait, M.; Hubert Joe, I.; Jayakumar, V.S. NIR-FT Raman, FT-IR and surface-enhanced Raman scattering spectra, with theoretical simulations on chloramphenicol. *J. Raman Spectrosc.* **2008**, *39*, 1772–1783, doi:https://doi.org/10.1002/jrs.2033.
